# Detection of Myositis Autoantibodies by Multi-Analytic Immunoassays in a Large Multicenter Cohort of Patients with Definite Idiopathic Inflammatory Myopathies

**DOI:** 10.3390/diagnostics13193080

**Published:** 2023-09-28

**Authors:** Anna Ghirardello, Mariele Gatto, Chiara Franco, Elisabetta Zanatta, Roberto Padoan, Luana Ienna, Nicoletta Gallo, Margherita Zen, Ingrid E. Lundberg, Michael Mahler, Andrea Doria, Luca Iaccarino

**Affiliations:** 1 Rheumatology Unit, Department of Medicine-DIMED, University Hospital of Padova, 35128 Padova, Italy; anna.ghirardello@unipd.it (A.G.); mariele.gatto@unipd.it (M.G.); chiara.franco@aopd.veneto.it (C.F.); elisabetta.zanatta@unipd.it (E.Z.); roberto.padoan@aopd.veneto.it (R.P.); luana.ienna@gmail.com (L.I.); margherita.zen@unipd.it (M.Z.); luca.iaccarino@unipd.it (L.I.); 2 Rheumatology Unit, Department of Clinical and Biological Sciences, Mauriziano Hospital, University of Turin, 10124 Turin, Italy; 3 Unit of Laboratory Medicine, Department of Medicine-DIMED, University Hospital of Padova, 35128 Padova, Italy; nicoletta.gallo@aopd.veneto.it; 4 Rheumatology Unit, Department of Medicine, Karolinska University Hospital in Solna, Karolinska Institutet, 171 77 Stockholm, Sweden; ingrid.lundberg@ki.se; 5 Werfen Autoimmunity, San Diego, CA 92131, USA; mmahler@werfen.com

**Keywords:** myositis-specific antibodies, myositis, laboratory tests, line blot, multi-analytic technology

## Abstract

Background: The usefulness of myositis-specific autoantibodies (MSAs) and myositis-associated autoantibodies (MAAs) for the assessment of idiopathic inflammatory myopathies (IIMs) is acknowledged, but laboratory standardization remains a challenge. We detected MSAs/MAAs by multi-analytic line immunoassay (LIA) and particle-based multi-analyte technology (PMAT) in a multicenter cohort of patients with IIMs. Methods: We tested the sera from 411 patients affected with definite IIM, including 142 polymyositis (PM), 147 dermatomyositis (DM), 19 cancer-associated myositis, and 103 overlap myositis syndrome (OM), and from 269 controls. MSAs/MAAs were determined by 16Ags LIA in all sera, and anti-HMGCR by ELISA in 157/411 IIM sera and 91/269 control sera. The analytical specificity of LIA/HMGCR ELISA was compared with that of PMAT in 89 MSA+ IIM sera. Results: MSAs/MAAs were positive in 307/411 (75%) IIM patients and 65/269 (24%) controls by LIA (Odds Ratio 9.26, 95% CI 6.43–13.13, *p* < 0.0001). The sensitivity/specificity of individual MSAs/MAAs were: 20%/100% (Jo-1), 3%/99.3% (PL-7), 4%/98.8% (PL-12), 1%/100% (EJ), 0.7%/100% (OJ), 9%/98% (SRP), 5.6%/99.6% (TIF1γ), 4.6%/99.6% (MDA5), 8%/96% (Mi-2), 1.5%/98% (NXP2), 1.7%/100% (SAE1), 4%/92% (Ku), 8.5%/99% (PM/Scl-100), 8%/96% (PM/Scl-75), and 25.5%/79% (Ro52). Anti-HMGCR was found in 8/157 (5%) IIM patients and 0/176 (0%) controls by ELISA (*p* = 0.007). Concordance between LIA/HMGCR ELISA and PMAT was found in 78/89 (88%) samples. Individual MSAs detected by LIA were associated with IIM subsets: Jo-1 with PM and OM, PL-12 with OM, Mi-2, TIF1γ, and MDA5 with DM, SRP with PM, and PM/Scl-75/100 with OM (*p* < 0.001 for all). Conclusions: Since MSAs are mostly mutually exclusive, multi-specific antibody profiling seems effective for a targeted clinical-serologic approach to the diagnosis of IIMs.

## 1. Introduction

Idiopathic inflammatory myopathies (IIMs) are systemic autoimmune diseases characterized, as for other connective tissue diseases, by peculiar serum autoantibodies [1,2,3]. The clinical picture of IIM primarily involves muscles, skin, lungs, and joints. According to current classification criteria [4,5,6], major IIM forms can be defined: dermatomyositis (DM), polymyositis (PM), inclusion body myositis (IBM), overlap syndrome with myositis (OM), and cancer-associated myositis (CAM). Anti-synthetase syndrome (ASyS) is a composite syndrome not definitely classified in any of those subsets, but patients with ASyS are usually classified into PM or OM. Autoantibodies towards intracellular proteins have been reported in 60–80% of patients with IIMs, depending on patient selection and laboratory detection methods.

Despite significant progress in biomarker research, novel diagnostic and prognostic biomarkers for myositis remain a relevant unmet need.

Over the last 15 years, increasing interest has been devoted to extensive autoantibody profiling in the diagnostic workup of IIMs [7,8].

Looking at diagnostic specificity, myositis-specific antibodies (MSAs) include mutually exclusive autoantibodies specific for the diagnosis of IIMs, whilst myositis-associated antibodies (MAAs), frequently present in association with MSAs, are not disease-specific.

Current classification criteria for IIMs [4,5] only include anti-Jo-1 antibody as an established biomarker for PM/DM, since laboratory diagnostics for the other MSAs are not widely applied, validated, and standardized [9,10,11]. Yet, a longstanding debate remains about the inclusion of the other MSAs/MAAs in the diagnostic workup of patients with IIMs [12].

Myositis autoantibodies target intracellular constituents, preferentially expressed in the cytoplasm rather than in the nucleus of the cell. Immune targets encompass highly conserved enzymes involved in key processes of cell biology, such as protein synthesis/transport, epigenetic regulation of gene transcription, innate immunity, muscle cell metabolism, and differentiation. They are constitutive intracellular ribonucleoproteins, but some of them are expressed in specialized tissues only, i.e., muscle-specific, as is the case of 3-hydroxy-3-methylglutaryl-Coenzyme A reductase (HMGCR), cytosolic 5′-Nucleotidase 1A (cN-1A), and Four-and-a-half-LIM-domain 1 (FHL1), or in response to peculiar stimuli, as for IFN-induced Melanoma Differentiation-Associated Antigen 5 (MDA5) (Table 1).

Each MSA is reliable to identify unique syndromes in the context of clinically defined PM, adult or juvenile DM, ASyS, and necrotizing or severe myopathy or sporadic IBM, representing a powerful tool to improve diagnosis, classification, and targeted treatment options [13,14,15]. Interestingly, intracytoplasmic enzymes involved in protein synthesis and transportation are the main targets of autoantibodies in PM, while autoantibodies associated with clinically different DM phenotypes target the nuclear transcription factors involved in epigenetic regulation of cellular homeostasis (Table 1).

Major MAAs, including anti-Ro52, anti-PM/Scl-75/100, anti-Ku, and anti-U1RNP, are reported in 20–50% of patients with IIMs and are thus helpful for diagnosis. They are mainly associated with overlap syndrome, a condition characterized by peculiar serologic and histologic features [16,17]. The most frequent MAA is anti-Ro52/TRIM21, found in about 30% of patients and mainly coexistent with anti-tRNA synthetase (ARS) or anti-MDA5 [17]. Anti-PM/Scl, anti-Ku, and anti-U1RNP are each found in about 4–15% of overlap syndrome patients [14,18]. Anti-PM-Scl or anti-Ku are most commonly associated with PM/DM and systemic sclerosis overlap syndrome, also called sclero-myositis, frequently complicated by interstitial lung disease [17]. Anti-U1RNP is frequent in PM/DM/SLE overlap syndrome and pathognomonic of mixed connective tissue disease. Recently, overlap myositis (OM) has been defined as a stand-alone entity, with peculiar phenotypes, serology, and muscle histopathology [18].

Actually, when IIMs are suspected, both MSAs and MAAs play a pivotal role in the assessment of diagnosis and prognosis [7], and therefore the treatment of the disease [18,19,20].

Due to wide antigen heterogeneity and poor expression in crude cell extracts, indirect immunofluorescence on HEp-2 cells or other routine tests are poorly sensitive and so inaccurate for myositis antibody detection. However, cytoplasmic staining should be looked for and reported in such patients.

The “reference” method to detect MSAs and identify new antibody reactivity is immunoprecipitation (IP) of radiolabeled proteins. Besides the discouraged/abandoned use of radioisotopes for protein labeling, several MSAs often show positive polypeptide bands of 140–150 kDa by IP, including anti-NXP2, anti-Mi-2, anti-MDA5, anti-TIF1γ, and anti-OJ antibodies, as it is difficult to distinguish these antibodies by molecular weight only [9]. IP is informative for research purposes, yet not for routine laboratory settings, being time-consuming, technically complex, and not applicable on large scales. Recently, complex (anti-OJ) or novel ARS have been investigated with new accurate methods, such as mass spectrometry coupled with IP or IP–Western blotting [21,22,23,24].

Over the years, commercial multi-parametric immunoassays have been developed for routine diagnostic work-up [25,26,27,28]. Line immunoassay (LIA) or dot-immunoassay, antigen-specific enzyme-linked immunosorbent assay (ELISA), multiplex quantitative immunoassays, i.e., ALBIA/Luminex technology, and fully automated particle-based multi-analyte technology (PMAT) represent reliable alternatives to cumbersome IP [29,30] (Table 2).

The harmonization and validation of methods on large multicenter cohorts has been recommended for diagnostic work-up [9,29,30].

The aim of our study was to investigate the MSA/MAA profile in a large multicenter cohort of patients with definite IIM by means of LIA, and assess MSA clinical correlates in the context of high *pre-test* probability of disease. In addition, the study aims to estimate the concordance (focus on analytical specificity) between LIA and PMAT for the detection of MSAs.

## 2. Materials and Methods

### 2.1. Patient Cohort

We carried out a retrospective cross-sectional study on consecutive Caucasian patients who received a diagnosis of definite IIM from January 2010 to September 2022 at the Rheumatology Unit, University-Hospital of Padova, Italy, and the Rheumatology Unit, Department of Medicine, Solna, Karolinska Institute, Stockholm, Sweden. We collected the sera from 411 adult patients affected with definite IIM according to the 2017 EULAR/ACR criteria [4,5,6], including 142 PM, 147 DM, 19 CAM, and 103 OM. Sixty-six out of one hundred and three OM (64%) had ASyS [31,32]. The sera from 57 sex–age-matched healthy subjects and 212 consecutive Caucasian patients with other diseases (11 non-autoimmune myopathy, 27 muscular dystrophy, 9 undifferentiated connective tissue disease, 90 systemic lupus erythematosus, 40 systemic sclerosis, 24 Sjögren’s syndrome, and 11 non-inflammatory myopathy or arthropathy) were tested as controls. Serum samples were stored at −80 °C until testing.

The study was approved by the Local Ethics Committee (Comitato Etico per la Sperimentazione dell’Azienda Ospedaliera di Padova, Prot. No. 2542P), and informed consent was obtained from all the subjects according to the Declaration of Helsinki.

### 2.2. Autoantibody Testing

MSAs and MAAs were determined by commercial line immunoassay (LIA, Euroline® Myositis profile 3, 16 Ags, Euroimmun, Lübeck, Germany; Mi-2α, Mi-2β, TIF1γ, MDA5, NXP2, SAE1, Ku, PM-Scl100, PM-Scl75, Jo-1, SRP, PL-7, PL-12, EJ, OJ, Ro52). In brief, purified antigens were coated as narrow lines on nitrocellulose chips, and immobilized on strips. After blocking of nonspecific binding sites with 5% nonfat milk in Tris-buffer/0.05% Tween 20 (TBS-Tween) for 30 min at room temperature (RT), each strip was incubated with 1:101 diluted serum samples in 5% milk/TBS-Tween for 30 min at RT. After three washes, the strips were incubated with 1:1000 diluted horseradish peroxidase-conjugated rabbit anti-human IgG (30 min at RT). After washing, bound enzyme was detected by reaction with 0.015% 4-chloro-1-naphthol 0.015% hydrogen peroxide in 16.7% methanol in TBS for 10 min at RT. Finally, the strips were washed in water and air-dried between sheets of filter paper, and the reactions were digitally scanned and semi-quantitatively measured by EuroLineScan®.

Anti-HMGCR antibodies were detected by commercial ELISA using human recombinant full-length HMGCR protein (QUANTA Lite HMGCR assay, Inova Diagnostics, San Diego, CA, USA) in 157/411 IIM sera and in 176/269 controls, according to the manufacturer’s instructions.

For comparison with a new PMAT, 89/411 IIM sera with at least one positive MSA (PL-7 = 4, PL-12 = 10, EJ = 1, HMGCR = 6, SAE1 = 4, Mi-2α/β = 13, TIF1γ = 18, MDA5 = 10, NXP2 = 4, and SRP = 19) were tested using the Aptiva™ instrument (Inova Diagnostics, San Diego, CA, USA) and the Aptiva Autoimmune Myopathy IgG™ reagents (Inova Diagnostics, San Diego, CA, USA, Research Use Only: PL-7, PL-12, EJ, Mi-2ß, TIF1γ, SAE, MDA5, NXP2, HMGCR, SRP54, PM-Scl-100), according to the manufacturer’s instructions [27,28]. Briefly, covalently bound antigens were coupled to paramagnetic particles that carry unique signatures and incubated with 1:100 diluted serum samples. After 9.5 min incubation at 37 °C, particles were washed and incubated for 9.5 min at 37 °C with anti-human IgG conjugated to phycoerythrin to label the bound autoantibodies. After the final wash cycle, the median fluorescence intensity (MFI) on the particles was captured using a digital imager and analyzed using proprietary algorithms to extract meaningful information for each analyte. Cut-off values of the different tests used, expressed as Arbitrary Units (AU)/mL, were those recommended by the manufacturers (11 AU LIA, 20 units HMGCR ELISA, 100 AU PMAT).

### 2.3. Statistical Methods

Data were statistically analyzed by SPSS 28.0 and GraphPad Prism 8.4. Differences in frequencies of dichotomous variables were analyzed by chi-square test or Fisher’s exact test. Pearson’s test was used to evaluate the correlation between MSA levels detected by LIA and PMAT assays. *p* values below 0.05 were considered statistically significant.

## 3. Results

The main demographic and clinical features of patients with IIMs have been reported in Table 3.

The main demographic and clinical features of control patients (*n* = 212) have been reported in Supplementary Table S1.

The prevalence of ANA > 1:80 and anti-ENA antibodies in IIM patients were, respectively, ANA 108/411 (26%), anti-ENA 110/411 (26.8%), comprising anti-Ro/SSA 106/411 (26%), anti-U1RNP 4/411 (1%), and anti-La/SSB 3/411 (0.7%).

**Figure 1:**
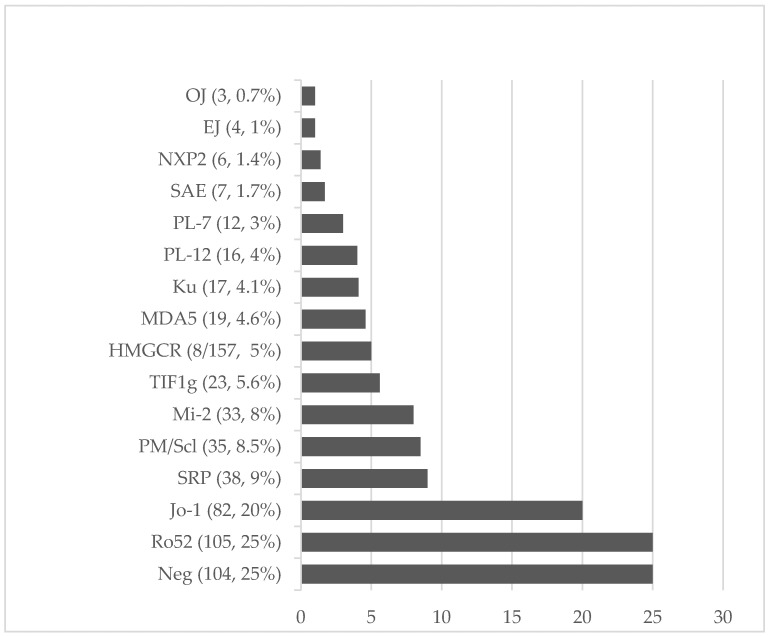
Frequency of myositis-specific antibodies (MSAs) and myositis-associated antibodies (MAAs) (number, percentual values) in 411 patients affected with definite idiopathic inflammatory myopathies (IIMs), detected by line immunoassay (LIA). Anti-HMGCR was determined in 157/411 IIMs by enzyme-linked immunosorbent assay (ELISA).

By LIA, MSAs/MAAs were positive in 307/411 (75%) IIM patients and in 65/269 (24%) controls (*p* < 0.0001, Odds Ratio (OR) 9.26, 95% CI 6.43–13.13, LR+ 3.091, PPV 0.82, NPV 0.66). MSA/MAA positivity was found in 14/57 (24.6%) healthy subjects and in 51/212 (24.0%) diseased controls. Anti-HMGCR was found in 8/157 (5%) IIM patients and 0/176 (0%) controls by ELISA (*p* = 0.007, OR 9.4, PPV 1.00, NPV 0.54). The prevalence of individual MSAs in IIM patients ranged from 0.7% for OJ to 20% for Jo-1 (for details see Figure 1).

MAAs were positive in 145/411 (35.3%) IIM patients and 77/269 (28.6%) controls (*p* = n.s.), of which anti-Ro52 was positive in 105/411 (25.5%) IIM patients and 56/269 (21%) controls (*p* = n.s.).

Representative images of positive results with anti-NXP2, anti-SRP, and anti-PL-7 serum samples by LIA are visualized in Supplementary Figure S1.

The specificity of individual MSAs/MAAs was: ≥99% (Jo-1, TIF1γ, PL-7, MDA5, PM/Scl-100, HMGCR, PL-12, Mi-2β, SAE), 98% (SRP, NXP2), 96% (Mi-2α, PM/Scl-75), 92% (Ku), and 79% (Ro52). No controls were positive for anti-EJ, anti-OJ, or anti-SAE (Table 4).

By LIA, multiple MSA positivity was found in 33/411 (8%) IIMs and in 17/269 (6%) controls (*p* = n.s.).

**Figure 2:**
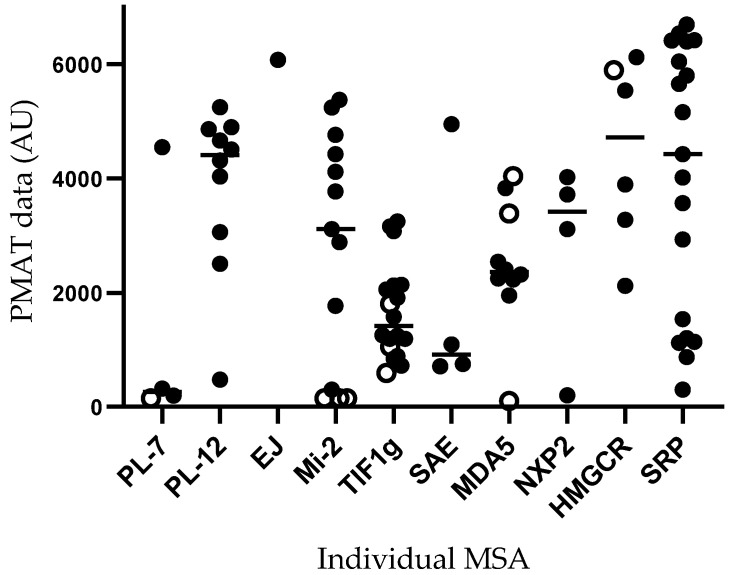
Individual myositis-specific antibody (MSA) positivity detected in 89 IIM sera by particle-based multi-analyte technology (PMAT): concordance/discordance with line immunoassay (LIA) and HMGCR ELISA results are depicted. Filled dots are concordant results between the methods, and open dots are discordant results between the methods.

The comparison between LIA/HMGCR ELISA and PMAT in 89 MSA-positive IIM patients (PL-7 = 4, PL-12 = 10, EJ = 1, HMGCR = 6, SAE1 = 4, Mi-2α/β = 13, TIF1γ = 18, MDA5 = 10, NXP2 = 4, and SRP = 19) showed concordance in 78/89 (88%) MSA-positive IIM sera (Figure 2).

The best concordance was obtained for SAE (4/4, 100%), NXP2 (4/4, 100%), SRP (19/19, 100%), EJ (1/1, 100%), PL-12 (10/10, 100%), followed by HMGCR (5/6, 83%), TIF1γ (15/18, 83%), Mi-2 (10/13, 77%), PL-7 (3/4, 75%), and MDA5 (7/10, 70%) (Figure 2). The discordant results between LIA and PMAT were the following: one ASyS (PL-7+ by LIA, negative by PMAT); one DM (Mi-2β+ by LIA, negative by PMAT); one PM (Mi-2α+ by LIA, negative by PMAT); one DM (Mi-2α/β+ by LIA, negative by PMAT); one DM (Mi-2α/β+ by LIA, negative by PMAT); one DM (negative by LIA, TIF1γ+ by PMAT, 592 AU); one DM (Mi-2α/β+ by LIA, TIF1γ+ by PMAT, 1804 AU); one OM (MDA5+ by LIA, negative by PMAT); one ASyS (negative by LIA, MDA5+ by PMAT, 4039 AU); one ASyS (EJ+ by LIA, MDA5+ by PMAT, 3388 AU); and one PM (PL-7+ by LIA, HMGCR+ by PMAT, 5900 AU). The PMAT assay we used did not include Jo-1, OJ, and Ku target antigens.

LIA semi-quantitative levels of positivity (AU/mL) and PMAT semi-quantitative values (AU/mL) in concordant positive samples (*n* = 78) were largely correlated (r = 0.56, 95% CI 0.39 to 0.70, R^2^ 0.32, *p* < 0.0001). As visualized in Supplementary Figure S2, a sort of bimodal distribution of LIA antibody levels was observed.

Multiple positivity was found in 8/89 (9.0%) samples by LIA and 3/89 (3.4%) by PMAT (*p* = n.s.) (Table 5).

**Figure 3:**
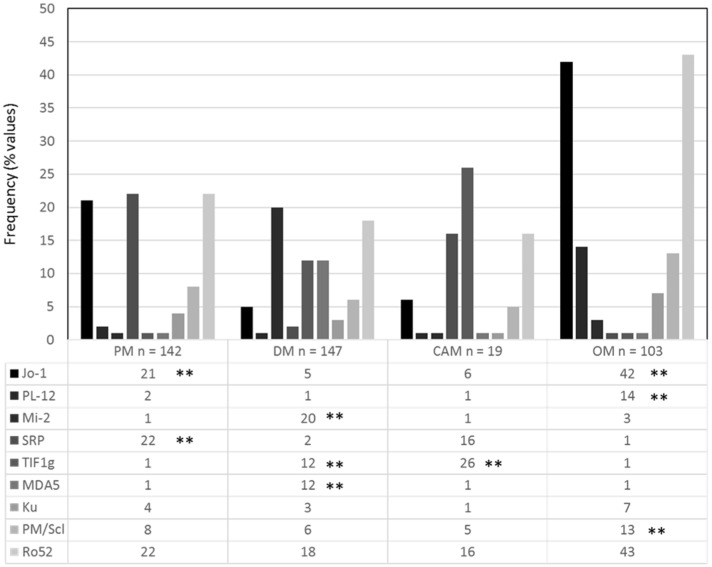
Clinical associations of myositis autoantibodies detected by line immunoassay (LIA) with IIM subgroups. The frequency of individual antibodies in the different IIM subsets is depicted. ** *p* < 0.001.

Individual MSAs, detected by LIA, were confirmed to be significantly associated with distinct IIM subgroups: Jo-1 with PM and OM (*p* < 0.001), PL-12 with OM (*p* < 0.001), Mi-2 with DM (*p* < 0.001), SRP with PM (*p* < 0.001), TIF-1γ with CAM and DM (*p* < 0.001), MDA5 with DM (*p* < 0.001), and PM/Scl with OM (*p* < 0.001) (Figure 3). As expected, anti-Ro52 and anti-Ku were not associated with distinct IIM subsets (*p* = n.s.).

## 4. Discussion

To date, the present study estimated the accuracy of multi-analytic LIA for the detection of MSAs/MAAs in the largest cohort of patients with definite IIM. High *pre-test* probability of disease was the condition specifically assessed in the study, as recommended by Bonroy C. et al. [30].

As MSAs are defined as “mutually exclusive”, their simultaneous detection in serum samples by multi-analytic testing is recommended for diagnostic purposes [33].

The difficulties of approaching MSA diagnostics by IP have prompted more feasible and automatized multi-specific assays. Concordance between IP and solid-phase immunoassays can be influenced by several factors: analytical accuracy, antigen purification and folding, proper equipment and procedures, and standardization limits. In solid-phase immunoassays, human recombinant full-length proteins as targets are expressed in different systems (*Escherichia coli*, Baculovirus-infected insect Sf9 cells or human HEK293 cells), immobilized on nitrocellulose chips, and specific antibody detection revealed by enzyme-linked chromogenic, chemiluminescent, or fluorescent detection systems. In contrast, IP typically investigates native ribonucleoprotein moieties in immuno-precipitates after liquid-phase incubation of serum with cell lysates, thus preserving conformational epitopes and antibody recognition. Nevertheless, IP’s complex and time-consuming nature largely limits its application in routine diagnostics.

LIA is the most widely used IVD (In Vitro Diagnostic Use) multi-analytic immunoassay for MSA detection, both in clinical and research settings (Table 2) [34].

Since the first validation of LIA for MSA [25], multi-specific testing has been implemented by virtue of novel targets and accuracy adjustment [26,27,28,29,34,35].

In the present study, extensive MSA/MAA profiling by third-generation LIA confirmed the diagnosis in 75% of patients with definite IIM, a frequency higher than 62%, found in 267 IIM patients by using second-generation LIA [25]. These promising results are mainly due to the high *pre-test* probability of IIM and the inclusion of anti-Ro52 and anti-HMGCR as clinically useful biomarkers. Ro52 protein is an E3 ubiquitin ligase involved in type I interferon responses, highly expressed in lymphoid tissues and lungs. Although not disease-specific, anti-Ro52/TRIM21 antibody is a highly frequent and established prognostic biomarker, being an independent risk factor for disease severity and relapse in connective tissue diseases, and of lung involvement in patients with IIMs [36,37,38,39]. In addition, the inclusion of anti-HMGCR has demonstrated high diagnostic accuracy due to mutually exclusive presence in 5% of IIM patients and 0% of healthy and disease controls. Anti-HMGCR and anti-SRP are independent biomarkers of immune-mediated necrotizing myopathy (IMNM), each accounting for 2/3 of IMNM patients, and involved in disease pathogenesis [40]. Novel automated assays for anti-HMGCR, including ELISA, chemiluminescence, and ALBIA, all demonstrated high diagnostic specificity and good agreement with IP [41,42,43,44].

Multi-parametric solid-phase assays could also have limitations, the main drawbacks being false positive results, e.g., anti-TIF1γ or anti-NXP2, multiple positivity, and cross-reactivity [45,46,47]. Regarding “multiple positivity”, according to our findings, it mainly occurs by LIA, and PMAT assay offers the great advantage of quantitatively measuring antibody level and identifying the highest antibody reactivity in samples with multiple positivities, as seen in Supplementary Figure S2.

Similar to other diagnostic areas, multi-parametric solid-phase assays could suffer from low reliability in low *pre-test* likelihood of IIM [48]. LIA and PMAT are both multi-analytic immunoassays that could give “false positives”, even in standard conditions, due to autoantibody polyclonality. Antibody titer quantification could be promising for this concern [49].

The high concordance between LIA and HMGCR/ELISA with PMAT, together with the valuable semi-quantitative estimation of antibody levels by PMAT (Figure 2 and Figure S2), represents a promising perspective for PMAT in the diagnostic work-up of IIMs, as recently suggested by Choi MY et al. [11].

The limitations of the study are that the LIA we used did not include HMGCR, and the “previous generation” PMAT panel we applied at the time of the study did not include both Jo-1 and OJ. Furthermore, the concordance between LIA and PMAT was investigated only in MSA-positive samples, in order to assess reciprocal analytical specificity and not clinical accuracy.

## 5. Conclusions

Multi-analytic detection of MSAs/MAAs is feasible in diagnostics, and crucial for supporting the diagnosis of IIMs in the context of high *pre-test* likelihood of IIM. Due to the mutual exclusivity of MSAs, extended antibody profiling is strictly effective for targeted clinical-serologic approaches to IIM clinical settings.

## Figures and Tables

**Table 1:** Myositis-specific antibodies and myositis-associated antibodies: target autoantigens and associated IIM phenotypes.

*Myositis-Specific Antibodies*			
Autoantibody	Target Autoantigen	Autoantigen Function	Associated Phenotype
Anti-ARS	Aminoacyl-tRNA synthetases: Jo-1, PL7, PL12, EJ, OJ, and others	Protein synthesis	PM/DM, ASyS
Anti-Mi-2	Nuclear DNA helicase	Transcription regulation	Benign DM
Anti-TIF1γ	Transcriptional Intermediary Factor 1-γ	Transcription and RNA metabolism	Severe DM, CAM
Anti-NXP2	Nuclear matrix Protein 2	Transcription repression p53 activation	JDM, adult DM, CAM
Anti-MDA5	Melanoma Differentiation-Associated protein 5 (*IFN-induced*)	Innate immune response to viral infection	Severe DM-ILD, CADM, JDM
Anti-SAE	SUMO 1 Activating Enzyme 1	Transcription regulation Post-translational modification	DM, CADM
Anti-SRP	Signal Recognition Particle: cytoplasmic translocation factor	Protein transport	IMNM
Anti-HMGCR	3-Hydroxy-3-Methylglutaryl-CoA Reductase (*muscle-specific*)	Cholesterol synthesis	IMNM (statin-induced)
Anti-cN-1A	Cytosolic 5′-Nucleotidase 1A (*muscle-specific*)	Muscle metabolism, RNA processing	sIBM
Anti-FHL1	Four-and-a-Half LIM protein 1 (*muscle-specific*)	Muscle-differentiation, sarcomere assembly	Severe myopathy with dysphagia
***Myositis-Associated Antibodies***			
**Autoantibody**	**Target Autoantigen**	**Autoantigen Function**	**Associated Phenotype**
Anti-Ro52	TRIM21 (*IFN-induced*)	Ubiquitination (IFNα regulation)	ASyS, PM, DM
Anti-PM/Scl	Ribonuclease (PM/Scl-100, PM/Scl-75 subunits)	Exosome complex	SSc-myositis overlap
Anti-Ku	DNA-binding protein	dsDNA break repair	SSc-myositis overlap
Anti-U1RNP	U1 ribonucleoprotein	Pre-mRNA splicing	PM/DM/SLE overlap, MCTD

Footnotes: ARS: anti-RNA synthetase; PM: polymyositis; DM: dermatomyositis, ASyS: Antisynthetase syndrome; ILD: interstitial lung disease; CADM: clinically amyopathic dermatomyositis; JDM: juvenile dermatomyositis, CAM: cancer-associated myositis; SUMO: Small-ubiquitin-like modifier; IMNM: immune-mediated necrotizing myopathy; sIBM: sporadic inclusion body myositis; SSc: systemic sclerosis; SLE: systemic lupus erythematosus; MCTD: mixed connective tissue disease.

**Table 2:** Analytical characteristics of current commercial multi-analytic immunoassays for the detection of myositis autoantibodies.

Procedure	Company	Target Autoantigens	Antigen Source	Serum Dilution	Cut-Off (Arbitrary Units/mL)	Results Interpretation	Laboratory Application (IVD/RUO)
Line immunoassay	EUROLINE® Euroimmun (Lübeck, Germany)	Mi-2α, Mi-2β, TIF1γ, MDA5, NXP2, SAE1, Ku, PM-Scl100, PM-Scl75, Jo-1, SRP, PL-7, PL-12, EJ, OJ, Ro-52	Recombinant, human; Affinity purified (Jo-1, MDA5)	1:101	11 AU	Digital, semi-quantitative	IVD
Dot-immunoassay	D-tek®/Alphadia (Mons, Belgium)	Jo-1, PL-7, PL-12, EJ, SRP-54, Mi-2, MDA-5, TIF1-γ, Ku, PM-Scl 100, Scl-70, SSA/Ro, SAE-1, SAE-2, NXP-2, OJ, KS, ZO	Recombinant, human	1:150	10 AU	Digital, semi-quantitative	IVD
PMAT	Autoimmune Myopathy IgG™ (Inova Diagnostics, San Diego, CA, USA)	Jo-1, PL-7, PL-12, EJ, OJ, Mi-2ß, TIF1γ, SAE, MDA5, NXP2, HMGCR, SRP54	Human recombinant	1:200	1 AU	Digital, quantitative	RUO
Line immunoassay	ImmcoStripe™ Myositis LIA, Trinity Biotech (Buffalo, NY, USA)	PM-Scl100, PM-Scl75, Ro-52, Jo-1, Mi-2, Ku, PL7, PL12, SRP54, U1RNP68, U1RNP A, U1RNP C, EJ, OJ	Not specified	1:100	Neg, Borderline, Pos	Visual, qualitative	IVD
Line immunoassay	Myositis Plus, Orgentec (Mainz, Germany)	AMA-M2, Jo-1, PM-ScI-100, PL-7, PL-12, Mi-2, Ku (p70/80), SRP, Rib-P	Affinity purified	1:100	Neg, Weak pos, Pos	Visual, semi-quantitative	IVD

Footnotes: IVD: In Vitro Diagnostic Use; RUO: Research Use Only; PMAT: particle-based multi-analyte technology; Pos: positive.

**Table 3:** Main demographic and clinical features of patients affected with definite idiopathic inflammatory myopathies (IIMs) (*n* = 411).

Features	Polymyositis (*n* = 142)	Dermatomyositis (*n* = 147)	Cancer-Associated Myositis (*n* = 19)	Overlap Myositis (*n* = 103)
Sex Ratio (F/M)	2.4 (102/42)	2.2 (101/46)	1.4 (11/8)	11.9 (95/8)
Age at diagnosis, years (mean ± SD)	52 ± 16	51 ± 18	64 ± 10	48 ± 16
Proximal muscle weakness	139 (98%)	125 (85%)	19 (100%)	89 (86%)
CK Increase	135 (95%)	126 (86%)	71 (69%)	17 (89%)
Skin disease	13 (9%)	124 (84%)	12(63%)	22 (21%)
Dyspnea	48 (34%)	19 (13%)	7 (37%)	73 (71%)
Interstitial lung disease	60 (42%)	35 (24%)	5 (28%)	73 (71%)
Arthralgia/Arthritis	86 (61%)	54 (37%)	9 (45%)	89 (86%)
Raynaud’s Phen.	48 (34%)	27 (18%)	2 (10%)	46 (45%)
Dysphagia	26 (19%)	34 (23%)	7 (37%)	15 (14%)
Fever	58 (41%)	42 (28%)	39 (38%)	5 (26%)

Footnotes: CK: creatin phosphokinase; SD: standard deviation.

**Table 4:** Diagnostic accuracy of myositis-specific antibodies (MSAs) detected by line immunoassay (LIA) in patients with idiopathic inflammatory myopathies (IIMs) and controls.

IIM MSA	Sensitivity n pos, % pos (95% CI)	Specificity n pos, % pos, (95% CI)	Odds Ratio (95% CI)
Jo-1	82/411 20 (16.4–24.1)	1/269 99.6 (97.9–99.9)	66.5 (12.5–673.8)
PL-7	12/411 2.9 (1.6–5.0)	2/269 99.3 (97.3–99.9)	4.0 (1.0–18.1)
PL-12	16/411 3.9 (2.4–6.2)	3/269 98.8 (96.7–99.7)	3.6 (1.1–11.7)
EJ	4/411 0.9 (0.4–2.4)	0/269 100.0 (97.9–100.0)	2.6 (0.4–32.3)
OJ	3/411 0.7 (0.2–2.1)	0/269 100.0 (97.9–100.0)	1.9 (0.3–25.7)
Mi-2	33/411 8.0 (5.8–11.1)	11/269 95.9 (92.8–97.7)	2.0 (1.0–4.1)
TIF1γ	23/411 5.6 (3.7–8.2)	1/269 99.6 (97.9–99.9)	15.9 (2.6–165.4)
MDA5	19/411 4.6 (2.9–7.1)	1/269 99.6 (97.9–99.9)	13.0 (2.3–136.3)
NXP2	6/411 1.5 (0.7–3.1)	5/269 98.1 (95.7–99.2)	0.8 (0.2–2.7)
SAE	7/411 1.7 (0.8–3.4)	0/269 100.0 (97.9–100.0)	4.7 (0.6–52.7)
SRP	38/411 9.2 (6.8–12.4)	5/269 98.1 (95.7–99.2)	5.4 (2.1–12.8)
HMGCR *	8/157 5.1 (2.6–9.7)	0/176 100 (96.8–100.0)	9.4 (1.4–104.9)

Footnotes: * Anti-HMGCR antibody was detected by ELISA in 157 IIMs and 176 controls. MSAs = myositis-specific antibodies; LIA = line immunoassay; IIMs = idiopathic inflammatory myopathies.

**Table 5:** A 1:1 comparison of multiple positive results between the line immunoassay (LIA) and the particle-based multi-analyte technology (PMAT) in IIM patients’ sera (*n* = 11).

Sample ID	IIM Patients’ Diagnosis	LIA Multiple Positivity	PMAT Multiple Positivity
43	Dermatomyositis	MDA5, PL-12, Ku	MDA5
50	Dermatomyositis	TIF1γ, PM/Scl-75	TIF1γ
55	Dermatomyositis	MDA5, Mi-2β	MDA5
64	Dermatomyositis	Mi-2α	Mi-2β, HMGCR
71	Dermatomyositis	TIF1γ, Jo-1	TIF1γ
89	Dermatomyositis	PL-7, PM/Scl-75	PL-7
73	Antisynthetase syndrome	OJ, NXP2, Ku	Neg
70	Polymyositis	Ku, PM/Scl-75	Neg
29	Dermatomyositis	Mi-2α/β	Mi-2β, TIF1γ, MDA5
86	Antisynthetase syndrome	EJ	MDA5, PL-12
56	Dermatomyositis	SAE, PL-12	SAE

## Data Availability

Not applicable.

## Supplementary Materials

The following supporting information can be downloaded at: https://www.mdpi.com/article/10.3390/diagnostics13193080/s1, Table S1: Demographics and clinical features of the control patients (*n* = 212); Figure S1: Representative image with positive results by line immunoassay: (a) anti-NXP2 serum; (b) anti-SRP serum; (c) anti-PL-7 serum; Figure S2: Correlation between line immunoassay (LIA) semi-quantitative positivity (AU/mL) and particle-based multi-analyte technology (PMAT) semi-quantitative positivity (AU/mL) in 78 concordant positive IIM sera.
