# Supplementary material for: Detection of Myositis Autoantibodies by Multi-Analytic Immunoassays in a Large Multicenter Cohort of Patients with Definite Idiopathic Inflammatory Myopathies
Source: Diagnostics (Basel). 2023 Sep 28;13(19):3080. doi: 10.3390/diagnostics13193080 (PMC10572214; doi:10.3390/diagnostics13193080)
Supplement: Supplementary file 1 [file diagnostics-13-03080-s001.zip › Table S1.pdf]

Supplementary Table S1: Demographics and clinical features of the control patients (*N* = 212)

**Features**	**SLE** *N* **= 90**	**UCTD** *N* **= 9**	**SSc** *N* **= 40**	**SS** *N* **= 24**	**Myopathy/ Arthopathy** *N* **= 49**
Sex Ratio (F/M)	5.9 (77/13)	8 (8/1)	12.3 (37/3)	2.4 (17/7)	0.7 (20/29)
Age at diagnosis, years (mean ± SD)	35 ± 10	34 ± 7	49 ± 5	53 ± 13	47 ± 10
Arthritis	17 (19%)	1 (10%)	10 (25%)	12 (50%)	12 (24.5%)
Skin disease	19 (21%)	1 (11%)	38 (95%)	8 (33%)	NA*
Raynaud's phenomenon	44 (49%)	6 (67%)	36 (90%)	4 (16.6%)	1 (2%)
Interstitial lung disease	4 (4.4%)	0 (0%)	17 (42.5%)	3 (12.5%)	0 (0%)
Muscle weakness	18 (20%)	2 (22%)	13 (32.5%)	7 (29%)	34 (69%)
Serositis	37 (41%)	1 (10%)	6 (15%)	6 (25%)	2 (4%)
Cytopenia	47 (52%)	1 (10%)	4 (10%)	7 (29%)	3 (6%)
GNF	43 (48%)	0 (0%)	1 (2.5%)	5 (21%)	0 (0%)
CNS involv.	7 (7.8%)	0 (0%)	0 (0%)	0 (0%)	0 (0%)
Thrombosis	18 (20%)	1 (10%)	1 (2.5%)	1 (4%)	1 (2%)
PNS involv.	7 (7.8%)	0 (0%)	2 (5%)	3 (12.5%)	0 (0%)

Footnotes: SLE: systemic lupus erythematosus; UCTD: undifferentiated connective tissue disease; SSc: systemic sclerosis; SS: Sjögren's syndrome; SD: standard deviation; NA: not applicable; * only psoriasis has been observed; GNF: glomerulonephritis; CNS: central nervous system; PNS: peripheral nervous system.
