# Supplementary figures and images for: Detection of Myositis Autoantibodies by Multi-Analytic Immunoassays in a Large Multicenter Cohort of Patients with Definite Idiopathic Inflammatory Myopathies
Source: Diagnostics (Basel). 2023 Sep 28;13(19):3080. doi: 10.3390/diagnostics13193080 (PMC10572214; doi:10.3390/diagnostics13193080)

Supplementary Figure S1

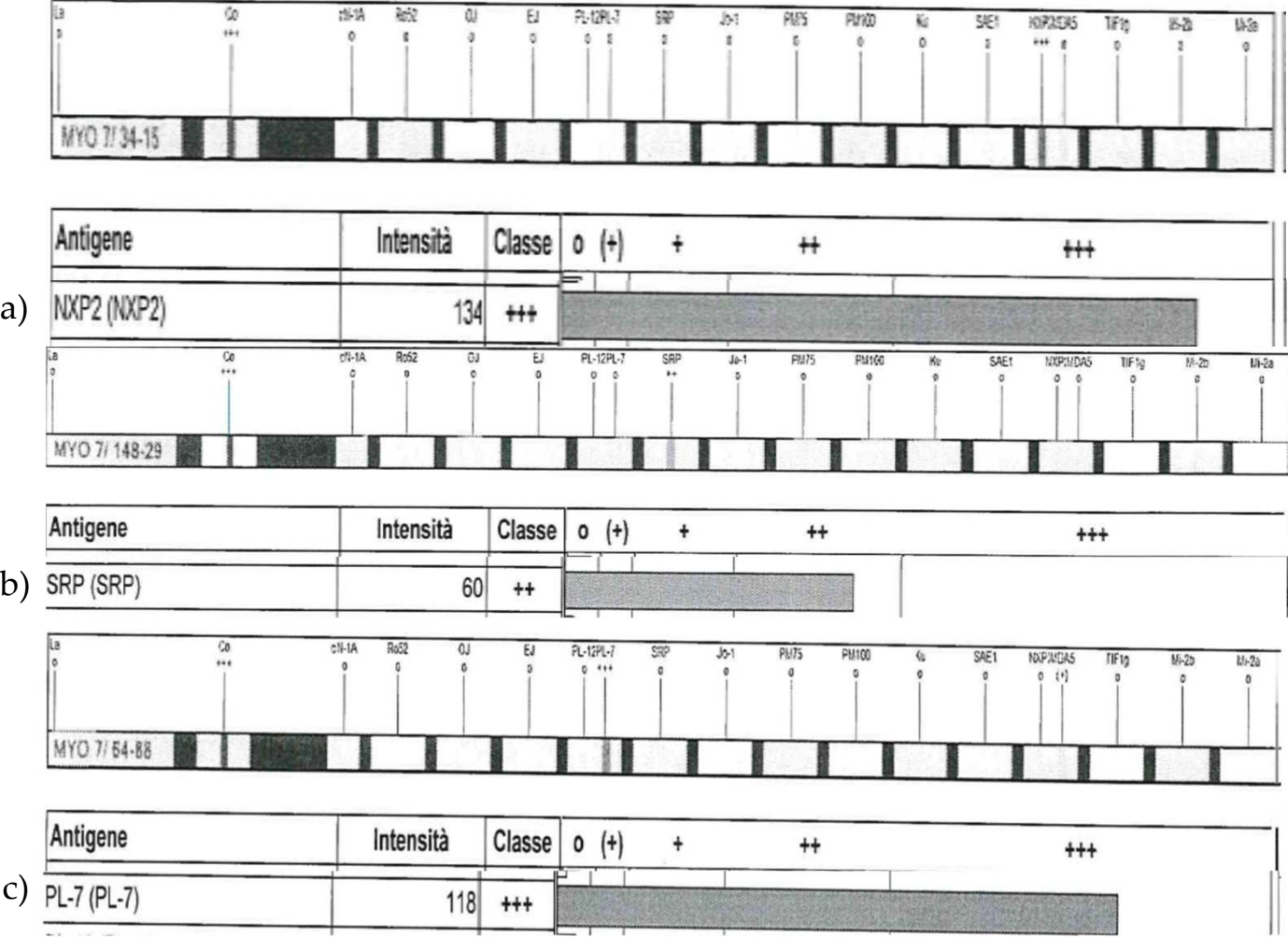

Supplement: Supplementary file 1 [file diagnostics-13-03080-s001.zip › Figure S1.pdf]

Supplementary Figure S2

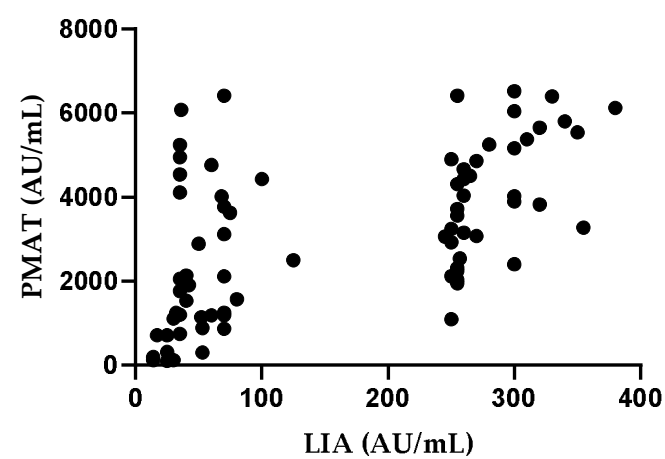

Supplement: Supplementary file 1 [file diagnostics-13-03080-s001.zip › Figure S2.pdf]
